# Energy metabolism as the hub of advanced non-small cell lung cancer management: a comprehensive view in the framework of predictive, preventive, and personalized medicine

**DOI:** 10.1007/s13167-024-00357-5

**Published:** 2024-04-08

**Authors:** Ousman Bajinka, Serge Yannick Ouedraogo, Olga Golubnitschaja, Na Li, Xianquan Zhan

**Affiliations:** 1 Medical Science and Technology Innovation Center, Shandong Provincial Key Medical and Health Laboratory of Ovarian Cancer Multiomics, & Shandong Key Laboratory of Radiation Oncology, Shandong Cancer Hospital and Institute, Shandong First Medical University and Shandong Academy of Medical Sciences, 440 Jiyan Road, Jinan, Shandong 250117 People’s Republic of China; 2 Predictive, Preventive and Personalised (3P) Medicine, University Hospital Bonn, Venusberg Campus 1, Rheinische Friedrich-Wilhelms-University of Bonn, 53127 Bonn, Germany

**Keywords:** Predictive preventive personalized medicine (PPPM / 3PM), Energy reprogramming, Non-small cell lung cancer (NSCLC), Metabolism, Proteoform, Proteoformics, Mitochondrial stress homostasis bioenergetics, Mitophagy, Phenotyping, Systemic effects, Multi-level diagnostics, Health risk assessment, Primary and secondary care, Suboptimal health, Health-to-disease transition, Flammer syndrome, Endothelin, Homocysteine, Individualized patient profile, Cost-efficacy, Health policy

## Abstract

Energy metabolism is a hub of governing all processes at cellular and organismal levels such as, on one hand, reparable vs. irreparable cell damage, cell fate (proliferation, survival, apoptosis, malignant transformation etc.), and, on the other hand, carcinogenesis, tumor development, progression and metastazing versus anti-cancer protection and cure. The orchestrator is the mitochondria who produce, store and invest energy, conduct intracellular and systemically relevant signals decisive for internal and environmental stress adaptation, and coordinate corresponding processes at cellular and organismal levels. Consequently, the quality of mitochondrial health and homeostasis is a reliable target for health risk assessment at the stage of reversible damage to the health followed by cost-effective personalized protection against health-to-disease transition as well as for targeted protection against the disease progression (secondary care of cancer patients against growing primary tumors and metastatic disease).

The energy reprogramming of non-small cell lung cancer (NSCLC) attracts particular attention as clinically relevant and instrumental for the paradigm change from reactive medical services to predictive, preventive and personalized medicine (3PM). This article provides a detailed overview towards mechanisms and biological pathways involving metabolic reprogramming (MR) with respect to inhibiting the synthesis of biomolecules and blocking common NSCLC metabolic pathways as anti-NSCLC therapeutic strategies. For instance, mitophagy recycles macromolecules to yield mitochondrial substrates for energy homeostasis and nucleotide synthesis. Histone modification and DNA methylation can predict the onset of diseases, and plasma C7 analysis is an efficient medical service potentially resulting in an optimized healthcare economy in corresponding areas. The MEMP scoring provides the guidance for immunotherapy, prognostic assessment, and anti-cancer drug development. Metabolite sensing mechanisms of nutrients and their derivatives are potential MR-related therapy in NSCLC. Moreover, miR-495-3p reprogramming of sphingolipid rheostat by targeting Sphk1, 22/FOXM1 axis regulation, and A2 receptor antagonist are highly promising therapy strategies. TFEB as a biomarker in predicting immune checkpoint blockade and redox-related lncRNA prognostic signature (redox-LPS) are considered reliable predictive approaches.

Finally, exemplified in this article metabolic phenotyping is instrumental for innovative population screening, health risk assessment, predictive multi-level diagnostics, targeted prevention, and treatment algorithms tailored to personalized patient profiles—all are essential pillars in the paradigm change from reactive medical services to 3PM approach in overall management of lung cancers. This article highlights the 3PM relevant innovation focused on energy metabolism as the hub to advance NSCLC management benefiting vulnerable subpopulations, affected patients, and healthcare at large.

**Supplementary Information:**

The online version contains supplementary material available at 10.1007/s13167-024-00357-5.

## Preamble

Energy metabolism is a hub of governing all processes at cellular and organismal levels such as, on one hand, reparable vs. irreparable cell damage, cell fate (proliferation, survival, apoptosis, malignant transformation, etc.), and, on the other hand, carcinogenesis, tumor development, progression and metastazing versus anti-cancer protection and cure. The orchestrator is mitochondria who produce, store and invest energy, conduct intracellular and system-relent signals decisive for internal and environmental stress adaptation, and coordinate corresponding processes at cellular and organismal levels [1, 2]. Consequently, the quality of mitochondrial health and homeostasis is a reliable target for the predictive approach in overall cancer management

- beginning with health risk assessment at the stage of reversible damage to the health followed by cost-effective personalized protection against health-to-disease transition (primary care of suboptimal health conditions of individuals predisposed to cancer development)
- and including targeted protection against the disease progression (secondary care of cancer patients against growing primary tumors and metastatic disease) [3].

Indeed, one can discriminate between several bioenergetic phenotypes and metabolic dependencies recently demonstrated for highly heterogeneous group of non-small cell lung cancers (NSCLC) [4]. Accoring to the research evidence presented, mitochondrial networks are organized into distinct subpopulations which in turn govern the bioenergic capacity of corresponding tumors. Further, mitochondrial homeostasis is interrelated with the innate immune sensing and Notch1-AMPK pathway influencing the quantity and characteristics of the pool of cancer stem-like cells. Corresponding mechanisms utilize specifically the hypermitophagy promoting metabolic adaptation and expansion of lung cancer [5]. In consensus, mitophagy is essential for glucose homeostasis and lung tumor maintenance [6], and an induced Pink1-Parkin pathway-mediated mitophagy promotes tolerance to toxic compounds and chemotherapy-resistence in patients with highly aggressive small cell lung cancers [7]. Indeed, dietary intervention is considered highly effective to modulate tumor microenvironment that, in turn, affects metabolism of malignant cells, their growth, and aggressivity in a multi-facted way [8]. On one hand, low glycemic diets may inhibit tumor progression by decreasing blood glucose and insulin levels [9–11]. On the other hand, under low nutrient supply in order to obtain nutrients, the malignant cells develop cannibalism in their microenvironment efficiently neutralizing the anti-tumor immune response and indicating poor prognosis in lung cancer [12].

Contextually, a precise metabolic phenotyping based on individualized patient profile is crucial to improve individual outcomes in overall lung cancer prevention and treatments. To this end, all relevant demographic, socioeconomical, clinical, non-clinical, and metabolic parameters have to be considered for individualized patient profile such as described elsewhere for other systemic disorders [13]. Specific clinically relevant phenotypes can be exemplified such as the Flammer syndrome [14]. Flammer syndrome phenotype (FSP) carriers have been described as being predisposed to metastatic disease, once the cancer is clinically manifested [15, 16]. In particular, disturbed microcirculation, psychologic distress, increased sensitivity to various stimuli (stress, drugs, etc.) and altered sense regulation such as pain, smell, and thirst perception, altered sleep patterns, systemic ischemic lesions and low-grade inflammation, low BMI, shifted metabolic profiles as well as frequently reported increased blood endothelin-1 (ET-1) levels, mitochondrial stress, impaired wound healing and existing pre-metastatic niches are characteristic for the FSP and highly relevant for poor individual outcomes of malignant transformation [17, 18]. To this end, systemic inflammatory responses are associated with poor overall survival of lung cancer patients [19]. Also high blood levels of the systemic vasoconstrictor ET-1 are associated with the lung cancer development [20] and poor survival of NSCLC patients—corresponding pahomechanisms are detailed in the literature including increased oxidative stress and cytosolic Ca^2+^ as well as promoted NSCLC cell proliferation in EGFR- and HER2-dependent manner [21]. Research data demonstrate that endothelin system is decisive for the phenotypic switches in the lung cancer, disease progression, and metastatic promotion [22]. In consensus, a physiologic stabilization of the ET-1 axis was demonstrated in preclinical studies as protective against lung cancer development [23].

Another clinically relevant phenotype is associated with alterations in one-carbon metabolism important for DNA synthesis and methylation. High plasma homocysteine (Hcy) and low folate levels have been associated with lung cancer development and progression [24], among other maliganancies which Hcy detection was suggested to be phenotypically relevant for [25]. Contextually, vitamin 6, 9, and 12 supplements seem to be protective against lung carcinogenesis [26] and supportive for the mental health intervention in treated NSCLC [27]. On the other hand, there are several clearly defined phenotypes in the population which suffer from enhanced Hcy levels in blood and therefore considered a target group to protect against lung cancer predisposition such as individuals

- with imbalanced diet and insufficient vitamin B 6, 9, and 12 intake
- diagnosed with disordered one-carbon metabolism
- diagnosed with obstructive sleep apnea associated with increased Hcy in blood [28], amongst others.

Above exemplified metabolic phenotyping is instrumental for innovative population screening, health risk assessment, predictive multi-level diagnostics, targeted prevention, and treatment algorithms tailored to personalized patient profiles—all are essential pillars in the paradigm change from reactive medical services to 3PM approach in overall management of lung cancers [29]. This article highlights 3PM relevant innovation focused on the energy metabolism as the hub to advance NSCLC management benefiting vulnerable subpopulations, affected patients, and healthcare at large.

## Non-small cell lung cancer in focus

As one of the main causes of cancer deaths globally, lung cancer is a significant health burden; thus, the need to understand the mechanisms underpinning the disease progression is imperative [30]. Based on its heterogeneous disease features, lung cancers are classified as small-cell lung carcinoma (SCLC), lung squamous cell carcinoma (LUSC), lung adenocarcinoma (LUAD), and large-cell carcinoma (LCC) [31]. Based on the cancer genome atlas (TCGA) project, there are 299 genes identified and 24 pathways/biological processes that drive the progression of lung tumors. In the recent cancer studies, the oncogenic alterations of the cellular metabolism are now understood as a strong effect, precipitated by the gene changes [32]. Cellular metabolism is associated with cancer driver mutations, and almost two thirds of cancers have glycolytic genes as part of the mutation. The conserved catabolic process that ensures cellular homeostasis as autophagy in lung cancers is an important tumor cell autonomous. The systemic autophagy sustains cancer cell metabolism and promotes immune evasion. Thus, an in-depth knowledge of this autophagy inhibition with its ability for non-tumor recovery is essential in cancer therapy [33]. It is almost a century since metabolic reprogramming (MR) through aerobic glycolysis was described by Otto Warburg. This comes with the pentose phosphate pathway (PPP) and citric acid cycle of the central carbon metabolism (TCA). Recently, cancer cell viability and growth is understood to be influenced by other factors besides TCA. For instance, vital nutrients and amino acids are strongly associated with MR in various forms of cancer [32, 34].

Among all these types of lung cancer, LUSC is the most common smoking-related NSCLC. Smoking can induce a metabolic switch, thereby altering the response to immunotherapy and reduces immune-checkpoint blockade (ICB) efficacy. The smoking-induced metabolic switch could lay foundations in treatment of non-smoker NSCLC patients as well [35]. For instance, polyunsaturated fat may reduce the risk of LUSC among smokers [36]. While up to 85% survival rate is known for stage 1, only 19% 1-year survival rate is established for distant metastatic disease (stage IV) [37]. Meanwhile, pulmonary adenocarcinomas form almost half of all lung cancer cases and are largely caused by smoking, specific gene mutations, and some occupational exposures [38]. In addition to the immune responses and epigenetic regulation linked to metastases, tumorigenesis and amino acids help maintain redox balance [39]. From the sex-specific lung cancer metabolic pathway study, global epigenetic changes are significant [40]. For instance, NRF2-antioxidant response element (ARE) pathway activation may increase cellular antioxidant defense, mitochondria reinforcement, and also MR. This may meet the increased energy demands of uncontrolled cell proliferation in lung cancers [41]. Activating transcription factor 3 (ATF3) as a stress-induced transcription factor is associated with the capacity of adipocyte and glucose metabolism [42]. As immune-evasive, cancers express immunomodulatory ligands. For instance, programmed death ligand-1 (PD-L1) reacts with programmed death receptor-1 (PD-1) while cluster of differentiation 80/86 (CD80/86) cytotoxic T-lymphocyte antigen-4 (CTLA-4) on tumor infiltration in metastatic NSCLC [43].

## Metabolic reprogramming in NSCLC

The growth, division, and survival of cancer cells depends on altered energy reprogramming. In lung cancers, metabolism-related subtypes can be used as biomarkers and help in both prognostics and treatment [43]. Tumor glycolysis has an inverse relationship with immune infiltration in cells [44]. To this end, specific immune infiltration can lead to novel findings for NSCLC. Through the regulation of energy metabolism, protein glycogen phosphorylase L (PYGL) is upregulated in various types of cancer. Since the mechanism involves mitotic function of cells, it could be a potential treatment for NSCLC [45]. Cancer MR elevates energy requirements and suppresses the human immune system thereby creating a microenvironment, suitable for the growth of tumor [46]. The primary energy metabolism of tumor cells is mitochondrial energy metabolic pathway (MEMP). Therefore, non-disruption to MEMP will only promote the progression of cancer, immune escape, and subsequently metastasis. The MEMP score can provide new guidance for immunotherapy, prognostic assessment, and also the development of anti-cancer through the DB0980 approach [47]. The compartmentalization of mitochondrial networks in NSCLC with distinct subpopulation enables the bioenergetic capacity for tumor growth [48]. To this end, one will need to study tumors with low rate of oxidative phosphorylation (OXPHOS^HI^) while monitoring the glucose influx and some structural remodeling of cristae.

As a heterogeneous disease with environmental and genetic parameters, NSCLC has a profound interplay between TME and of the metabolic activities of the tumor and also immune response of the host cells. Cancer cells rewire their metabolism to ensure the continuous growth, invasiveness, and metastatic properties and promote adaptive resistance to chemo-radiotherapy. MR in cancer cells include proliferation, migration, angiogenesis, invasion, and giving distinct phenotypic features to cancer cells. The oncometabolites induced by metabolic disorders in cancer cells promote the growth of cancer and subsequently forming a vicious circle. One key of interest that might serve as a therapeutic strategy is the metabolite sensing mechanisms of nutrients and their derivatives [49]. Through the activation of processes that are studied to support survival of cell growth, proliferation, and growth, MR takes an active role in tumorigenesis in TME. Immunotherapy’s effectiveness in NSCLC is based on targeting and manipulating metabolic pathways [50, 51]. MR is affected by DG1 that could inhibit NSCLC proliferation. As a thymidylate synthase inhibitor, DG1 is promising for NSCLC angiogenesis treatment [52]. Another potential treatment strategy could be miR-mediated mechanisms in reprogramming sphingolipids. miR-495-3p can reprogram sphingolipid rheostat by targeting Sphk1, thus induces lethal mitophagy that suppresses NSCLC tumorigenesis [53]. Amino acids, carbohydrates, and nucleotides are metabolic super pathways that are beyond the Warburg effect, thus contributing to clinical significance [54].

LUSC therapy can be obstructed by receptor tyrosine kinase (RTK)-RAS inhibition due to the loss of the epigenetic modulator, KMT2D. This is one of the most frequently mutated genes in LUSC and regulates oncogenesis [55]. The clinical relevance of the distinct genomic landscape of KRAS oncogene in NSCLC might reveal specific therapeutic interventions [56], considering high levels of adenosine as a typical characteristic of tumor immune microenvironment (TIME) while having significant impact on both immune response and tumor cell growth. A2 receptor antagonist could be a potential therapy strategy in NSCLC [57]. Transcription factor EB (TFEB) gene can upregulate Siglec-15 expression, then bind to Ldha and Hk2 promoters thereby enhancing glycolytic influx in NSCLC cells. Inhibiting TFEB is found to improve the anti-PD-1 therapeutic efficiency in obese mice; thus, TFEB is a biomarker in predicting immune checkpoint blockade [58].

## Inhibiting the synthesis of biomolecule

### Nucleotide synthesis

Due to its multiple biological processes in tumor cells, proptosis-related LncRNA signature can assess immune function and drug sensitivity in NSCLC and thus serve as a predictor to prognosis [59]. The regulation and preservation of mitochondrial quality by autophagy in NSCLC helps fatal nucleotide pool depletion and prevent energy crisis [60]. For instance, a redox-related lncRNA prognostic signature (redox-LPS) validated for NSCLC patients has provided strategies for precision medicine and clinical decision-making [61]. The role of redox-associated genes should be studied for NSCLC as a prognostic model [32]. In addition, FTX, LINC00472, PSMA3-AS1, and SNHG14 are the 4 critical glycolysis-related lncRNAs [62]. Through the regulation of 22/FOXM1 axis, lncRNA NNT-AS1 plays a key role in carcinogenesis in NSCLC, thus a novel pathogenesis and a paradigm shift into the therapeutic target for these forms of cancer [63]. The immunotherapy of LUSC is influenced by tumor-infiltrating immune cells (TIICs), pathological stage, metabolism, and also the survival of patients [64]. In regulating genes and identifying prognostic indicators of LUSC, T DNA methylation data and TCGA-derived miRNA/mRNA sequencing are imperative [65]. The genomic process that can disrupt genes thereby leading to tumor occurrence somatic long interspersed element-1 (*LINE-1-FGGY*) is a potential therapeutic target and a prognosis predictive biomarker for LUSC local immune evasion [66].

### Ribosome biogenesis

Due to its role in tumorigenesis, ribosome-targeted therapy is a promising approach for treating patients with cancer. The tumor heterogeneity with pathological staging global metabolic parameters are related [35]. In the light of the upregulation of glucose-requiring hexosamine biosynthetic pathway (HBP) and the coat complex II (COPII), LUAD and LUSC subtypes can be distinguished based on their adaptive mechanisms of the TME even in glucose-deprived conditions. Herein, high expression of GFAT1 (HBP rate-limiting enzyme) is associated with wild-type EGRF activation [67]. The flavone cirsilineol can inhibit the proliferation of NCIH-520 cells through the induction of ROS-mediated apoptosis [68]. Dual-energy CT has an improved diagnostic for lymph node metastasis in patients with NSCLC [69]. A novel crystal (E)-4-(4-methylbenzyl)-6-styrylpyridazin-3(2H)-one (E-BSP) is a potential inhibitor of LUSC [70], and radiomic features can identify clinical and core signaling pathways of LUSC [71].

### Protein synthesis

The tumor protein PD-L1 interaction with the immune system is blocked by pembrolizumab thus enabling immune response in various types of cancer [72]. Through epithelial-mesenchymal transition (EMT), miR-607 and calcium-activated nucleotidase 1 (CANT1) pair is key for LUSC therapeutic strategies [73]. Moreover, Rb protein can be used for independent prognostic factors in early-stage NSCLC [74]. The p45 protein is predicted to be associated with malignant transformation via p36cyclinD1 regulation [75]. In the human LSCC line called Ben, parathyroid hormone-related protein (PTHrP) production can be regulated with IL-6-treated cells and PTHrP is influenced by both insulin-like growth factors I and II (IGF-I, IGF-II) [76]. Chemokine receptor CXCR4 induced SDF-1/CXCR4 axis for NSCLC patients may lead to important implications [77]. EpCAM and TROP2 gene overexpressions were found to be correlated with NSCLC [78]. Due to the phosphorylation of eukaryotic translation initiation factor 4E (eIF4E) binding protein (4E-BP1), p-4E-BP1 Thr37/46 had a poor prognostic significance in NSCLC [79].

Squamous cell carcinoma antigen 1 (SCCA1) can sensitize cells to endoplasmic reticulum (ER) stress through the activation of caspase-8 independent of the death receptor apoptotic pathway [80]. The EGFR family member of HER3 blocking antibody, U3-1287/AMG888, when complimented with radiotherapy could reduce cell and tumor growth and thus will increase lung tumor DNA damage and cell death [81]. However, since a study on East Asians and Western populations expressed distinct EGFR gene and protein, histology and staging in NSCLC should be analyzed for any large cohort study [82]. The lack of PIAS3 protein expression post-translational modifications in SCC made PIAS3 a potential therapeutic molecule that will target the STAT3 pathway in NSCLC [83]. Expression of apoptosis blocking bcl-2 protein predicts a poor prognosis for radiation-treated NSCLC patients [84]. Bronchoalveolar lavage (BAL)-exosomal human aspartyl β-hydroxylase (ASPH) is a potential biomarker for NSCLC diagnosis [85].

MicroRNA-26a (miR-26a) as an anti-oncogene regulates tumorigenic properties of EZH2 in human lung carcinoma cells [86]. Moreover, EZH2 can promote tumor progression via regulating VEGF-A/AKT signaling in NSCLC [87]. Src kinase inhibition induced by dasatinib is effective againtst cisplatin resistance [88]. Insulin-like growth factor binding protein-3 (IGFBP-3) with its molecular framework can serve as a new line of antiangiogenic cancer drugs [89]. Fascin actin-bundling protein 1 (FSCN1) and protein tyrosine phosphatase receptor type F (PTPRF) promote tumor progression in LUSC [90]. The CXCL12/CXCR4 produced by Prx1+ mesenchymal cells can be a target to eradicate parenchymal leukemia stem cells (LSCs) in acute myeloid leukemia (AML) [69]. Both respiratory chain genes and mitochondrial ribosomal protein can impact in vivo tumor growth. This was seen in a context-specific manner and differential impacts on both primary and metastatic tumors [91]. Auranofin-induced cell death due to increased ROS levels and glutathione (GSH) depletion is strongly associated with oxidative stress in lung cancer cells [92].

### Summary of inhibiting the synthesis of biomolecule

**Fig. 1:**
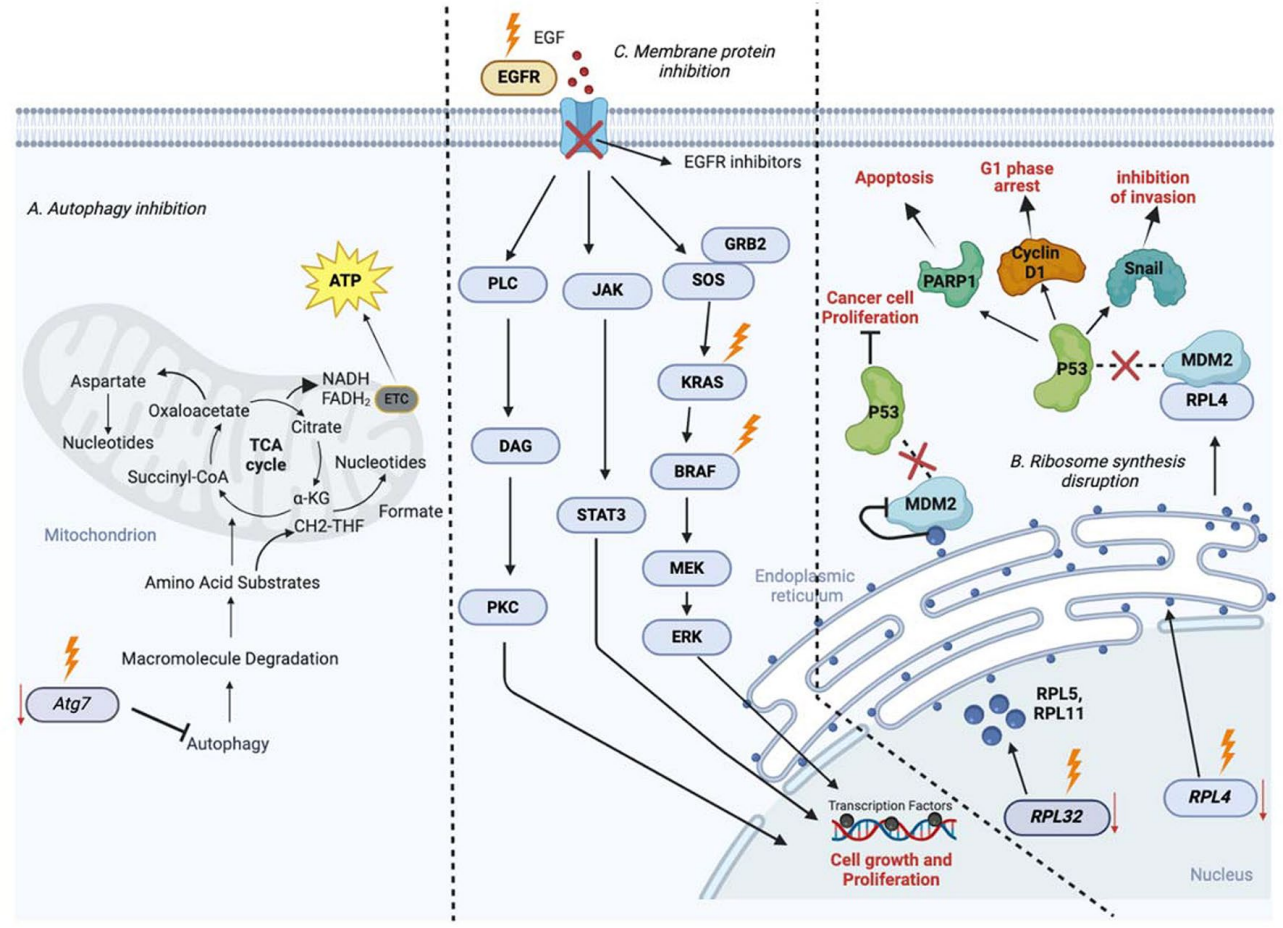
Inhibition of the synthesis and function of biomolecules in NSCLC. **A** Autophagy inhibition. **B** Ribosome synthesis disruption. **C** Membrane protein inhibition. BRAF, B-Raf proto-oncogene; EGFR, estimated glomerular filtration rate; FAO, fatty acid oxidation; HER4, human epidermal growth factor receptor 4; JAK-STATs, Janus kinases-signal transducer and activator of transcription proteins; KRAS, Kirsten rat sarcoma virus; MDM2, mouse double minute 2 homolog; PARP14, a member of the Poly (ADP-ribose) polymerase (PARP) family; RPL4, ribosomal protein L4

Autophagy recycles macromolecules to provide mitochondrial substrates for nucleotide synthesis and energy homeostasis. *Atg7* deficiency/inhibition reduces Kras^G12D^-driven NSCLC proliferation and tumor burden by preventing autophagy, which causes impaired mitochondrial respiration and fatty acid oxidation (FAO) leading to metabolic impairment (Fig. 1A). The downregulation of ribosomal protein L4 (RPL4) inhibits the development of NSCLC cells by disrupting the MDM2-P53 pathway and altering PARP1/Snail/cyclin D1 expression with lead to apoptosis, invasion inhibition, and G1-phase arrest. RPL32 is overexpressed in lung cancer and is associated with a bad prognosis. RPL32 knockdown causes ribosomal stress and hampers rRNA maturation. RPL5 and RPL11 recognize stress and transfer from the nucleus to the nucleoplasm where they bind with MDM2, a key p53 E3 ubiquitin ligase, resulting in p53 accumulation and suppression of cancer cell proliferation (Fig. 1B). The transmembrane glycoprotein known as EGFR (HER4) interacts to ligands, and activates intracellular signaling pathways such as JAK-STAT, PLC-gamma, PI3K/Akt, and MAPK, which are involved in cell proliferation, differentiation, migration, and death. Thus, inhibiting this protein induces NSCLC cell death. Notably, KRAS and BRAF can also be targeted to hamper lung cancer progression (Fig. 1C).

## Blocking common NSCLC metabolic pathways as anti-NSCLC

### Glutamine metabolism synthesis

With oncogenic mutations, the metabolism of glutamine (glutaminolysis) is essential for the proliferation of cancer cells. This is extensively studied with BRAF and KRAS mutation or active c-MYC [93]. It is established that a deficiency in glutamine can induce AMPK-mediated CHKα2 S279 phosphorylation. This in turn promotes the binding of CHKα2 to lipid droplets, thereby recruiting autophagosomes and cytosolic lipase ATGL. Subsequently, NSCLC tumor survival and proliferation is facilitated through lipolysis of lipid droplets [94]. A deletion of glutamine means inhibiting glutamine transporter (SLC1A5) expression that reduces cellular glutamine uptake in NSCLC cells. Therefore, the combination of SLC1A5 inhibition with almonertinib and/or V9302 is promising for the induction of apoptosis via autophagy inhibition in NSCLC [95].

Osmundacetone (OSC) in mitochondrial energy metabolism in NSCLC cells suppresses the development of tumor and proliferation. This effects in downregulating GLUD1 to inhibit glutamine metabolic axis and thus serves as an anti-cancer metabolic modulator in personalized chemotherapy of NSCLC [96]. Knockdown of angiopoietin-like protein (ANGPTL) 4 as a key regulator for lipid and glucose metabolism affects the glutamine consumption. This inhibits tumor energy metabolism and fatty acid oxidation in NSCLC [97]. KEAP1/NRF2 pathway (KLK) tumors exhibit an increased expression of genes that are involved in glutamine metabolism in *KRAS*-mutant NSCLC [98]. Kruppel-like factor 2 (KLF2) may decrease glutamine levels and thus inhibit energy metabolism in NSCLC [99]. NF-κB can upregulate glutamine-fructose-6-phosphate transaminase 2 (GFPT2), thereby promoting migration in NSCLC. Therefore, modulating GFPT2 is crucial in targeted therapy to combat disease progression for NSCLC [100]. Tumor necrosis factor receptor-associated protein 1 (TRAP1) inhibitor increases glutamine synthetase (GS) activity, glutamine auxotrophic of NSCLC [101]. Moreover, glutamine metabolism in cisplatin-resistant cells is mostly required for nucleotide biosynthesis. This metabolic vulnerability of cisplatin-resistant cancers target nucleoside metabolism in NSCLC [102].

### Lipid biosynthesis

Tumor cells are studied to co-opt adipocytes in the TME, thereby converting them into cancer-associated adipocytes (CAA). The enlargement of cancer cells and adipocytes must ensure the bi-directional signaling that is symbiotic between the two. Lung cancers stimulate lipolysis in adipocyte and fatty acid (FA) uptake from the adipose tissue. This FA is used for energy metabolism (β-oxidation), lipid-derived cell signaling molecules (which are linolenic acid and derivatives of arachidonic), and membrane synthesis. Therefore, approaches in blocking lipid associated metabolic pathways in lung cancer could lead to a profound strategy for lipid-enriched lung cancer TME [103]. In the pre-metastatic lung, neutral lipids are accumulated by neutrophils through the adipose triglyceride lipase (ATGL) activity. This is facilitated through prostaglandin E2-independent manners. While inhibition of this ATGL activity has been shown to alter breast tumor lung metastatic and neutrophil lipid profiles in mice models, it could be studied for NSCLC using high-throughput sequencing [34]. Lipid makers can serve as biomarkers using blood tests for early diagnosis of LUSC [104].

High-dose dexamethasone (DEX)-inhibited tumor progression is only activated by M1-like tumor-associated macrophages (TAMs) but also limit the uptake of glucose and lipids. This subsequently suffocates the cells through blocking the energy supply of cancer cells. Therefore, activated M1-like TAMs with inefficient lipid and glucose metabolism can delay tumor cell growth and promote apoptosis [105]. Moreover, blockade of nanodiamond-doxorubicin conjugates (Nano-DOX)-induced PD-L1 in the lung cancer cells enhanced activation of tumor-associated macrophage (TAM)-mediated anti-tumor response [106]. Low-molecular-weight β-glucan (LMBG) confers antitumor activity via a non-specific immune response [107]. Impaired muscle protein synthesis and fat metabolism through suppressed rampamycin (mTOR) signaling in NSCLC will give some etiology of the cancer type [42]. Among the recent clinical trials, mTOR inhibitors, glutaminase inhibitors, and anti-PD-L1 therapy in lung cancer patients have clinical significance [108]. After surgery, SNPs in de novo lipogenesis (DNL) genes are prognostic markers for NSCLC [37].

Ferroptosis suppressor protein 1 (FSP1) confers protection against the glutathione peroxidase 4 (GPX4), which is a phospholipid hydroperoxide-reducing enzyme. Moreover, GPX4 inhibitors can trigger ferroptosis, an iron-dependent form of necrotic cell death, which is marked by oxidative damage to phospholipid [109]. The role GPX4 expression in preventing iron-dependent lipid peroxidation-mediated cell death (ferroptosis) could be used as therapeutic for LUSC as it is studied to inhibit Mycobacterium tuberculosis-induced necrosis [110].

### Citric acid cycle (TCA)

Glucose is burnt by tissue via TCA cycle to CO_2_ under aerobic conditions or metabolized anaerobically via glycolysis to lactate. Lactate is a potential source of nutrients to the tumor cells, making TCA substrate primary circulating lactate in most tumors and tissues [111]. In the human NSCLC TCA cycle, lactate and not glucose predominates, making lactate the bona fide energy source for this type of cancer. Extensive metabolites of TCA cycle were seen with ^13^C-lactate infusing human NSCLC patients [112]. However, as opposed to the common belief (hypermetabolic), lung solid tumors produce ATP at a slower rate especially with protein synthesis downregulation for pancreatic cancer. This calls for a new approach to glycolysis flux with low TCA flux and ATP production [113]. Even primary clear cell renal cell carcinoma (ccRCC) show the lowest enrichment in TCA cycle intermediates and higher glycolytic intermediates [114].

### Tumor glycolysis blocking

It is strongly established that cancer cells utilize aerobic glycolysis (“the Warburg effect”) in order to produce energy. This concept is complemented with enhanced tumor reliance on oxidative metabolism through cisplatin resistance (CR) tumors [115]. The aerobic glycolysis that favors the growth of cancer is through oncogenic signaling pathway programming of cancer cell metabolism. This promotes the evasion of immunosurveillance, and through T cell function regulators, this oncogene-induced MR is linked with immune escape. For instance, increased glycolysis is correlated with dysregulation in lung cancer, called Notch1 signaling, and Notch1/TAZ axis modulation is crucial for lung aerobic glycolysis [116]. It is apparent that tumor metabolites such as tryptophan catabolism (kynurenine pathway) are effectors of immune cells during acquisition of CR resistance in the TME. Thus, targeting CR cells, the changes in metabolism in correlation with immune cells in the TME will provide rooms for CR-resistant therapeutic strategies [117]. The need to explore glycolysis-related genes (GRGs) are associated with tumor immune prognosis of NSCLC patients, and the activation of STING signaling in dendritic cells (DCs) is imperative [118].

Tumor glycolysis is studied to be associated with the efficacy of adoptive T cell therapy (ACT), and this could be a candidate targeted for combinatorial therapeutic intervention for NSCLC [119]. The mechanisms by which individual peroxiredoxin (PRDX) controls LUSC in complementation of PRDX oxidation state, configuration, the client proteins [120], and transcription factor (TF) regulatory network for NSCLC should be explored [121]. In CRC cells, the energy consumption of mitochondria and glycolysis of ATP is actualized with the help of myeloid cells or novel protein prokineticin 2 (Bv8) [122]. Beside PI3K signaling pathway, VEGF/VEGFR signaling, and CDK4/6 pathway, all of KEAP1/NRF2 pathway, FGFR1, and EGFR signaling pathway in addition to SOX2 and TP63 differentiation makers for chromosome 3q. are therapeutic potential for NSCLC [123, 124]. Through ERK/c-Myc pathway, artemisinin derivatives DHA and AS can inhibit NSCLC, and thus this could be a regulatory strategy for tumor glucose metabolism [125]. Since the lactate-rich characteristic of NSCLC is found to provide an exploitable property that improve NSCLC outcomes, the design can make new therapeutic strategies when integrated with conventional therapies such as carnitine palmitoyltransferase (CPT) system [126, 127]. The comprehensive analysis of NPM1 gene in LUAD showed that the expression of NPM1 gene is strongly correlated with five glycolysis-related genes (ENO1, HK2, LDHA, LDHB, and SLC2A1) and one m6A modifier-related gene (YTHDF2). Thus, NPM1 is a potential prognostic biomarker that is involved in immune infiltration of LUAD and also associated with m6A modification and glycolysis [128].

The tumor suppressor gene called liver kinase B1 (LKB1) or serine/threonine kinase 11 (STK11) is largely detected in NSCLC. For instance, an improved outcome of NSCLC patients treated with chemotherapy was based on the redox homeostasis and energy depletion due to lost of LKB1-AMPK signaling [129]. Moreover, LKB1/AMPK signaling axis can be compromised by LKB1 through aurora-A-mediated phosphorylation and thus enchances the growth and migration of NSCLC [130]. Of note, AMPK-related kinases are a master regulator of cell survival during stress conditions. Inactivation of *STK11/LKB1* leads to a reduced density of infiltrating cytotoxic CD8^+^ T lymphocytes, neutrophil-enriched TME, lowered PD-(L)1 expresion, and inert TIME [131]. In addition, dichloroacetic acid (DCA) was found to synergically affect SIRT2 inhibitor, Sirtinol, and AGK2 in enhancing anti-tumor efficacy in NSCLC [132].

In designing targeted therapeutic drugs for NSCLC based on the dysregulated signaling and metabolic pathways, LKB1-deficient is crucial. The loss of LKB1 expression can alter mitochondrial dysfunction and energy metabolism of the cells. One such treatment that confuses cellular response and thus resulting to impaired synthesis of ATP homoeostasis is erlotinib treatment. This can induce apotosis in LKB1-deficient cells in addition to inhition of cell growth and blocking of rapamycin signaling [133]. FBXO22 can mediate Lys-63-linked LKB1 polyubiquitination thus inhibits kinase activity of LKB1. Since overexpression of FBXO22 promotes NSCLC cell growth, inhibiting LKB1-AMPK-mTOR signaling is a potential therapeutic target [134]. Phosphoglycerate dehydrogenase (PHGDH) de novo serine synthesis pathway is a hallmark of metabolic adaption in carcinogenesis. For instance, an increased expression of PHGDH was seen in protein, and mRNA of NSCLC cells makes it a potential therapeutic strategy [135].

### β-Oxidation

Mitochondria fatty acid β-oxidation (FAO) alters cell fate decisions [136]. This type of energy metabolism of β-oxidation enters through binding proteins and specific fatty acid receptors [137]. Mouse model of Li-Fraumeni Syndrome revealed that fatty acid oxidation slows the free survival of cancers [138]. Beta-oxidation as an essential process in energy metabolism is a good source of acetyl-CoA, which serves as a substrate for protein acetylation, ketone body synthesis, phase II detoxification, and cholesterol synthesis [139]. Among the identified energy reprogramming, mitochondrial trifunctional protein (MTP) plays an important role in FAO [140]. Viperin-mediated metabolic alteration can inhibit FAO to enhance progression of cancer [141]. Diosbulbin B (DIOB)-mediated inhibition of FAO is one of its molecular mechanisms [142]. Tumor infiltrating myeloid-derived suppressor cells (MDSC) leads to upregulation of key FAO enzymes, increased oxygen consumption rate, and increased mitochondrial mass. So, once this FAO is inhibited pharmacologically, it will block its function in T-MDSC and also block the immune inhibitory pathway, thereby producing inhibitory cytokines. Combining FAO inhibition with low-dose chemotherapy can completely inhibit T-MDSC immunosuppressive effects [136]. Moreover, the blocking of FAO mithocondrial pathway with chemotherapy for NSCLC can give an enhanced anti-tumor effect [143].

Both for in vitro and in vivo peroxisome proliferator-activated receptor gamma (PPARγ) with its function in tumor suppressing can transactivate genes for β-oxidation [144, 145]. Mutant KRAS promotes FAO through acyl-coenzyme A (CoA) synthetase long-chain family member 3 (ACSL3) in lung cancer cells in an ACSL3-dependent manner [146]. Phytopharmaceutical mangiferin (MGF) targeting FAO metabolism can inhibit tumor, metastasis, and angiogenesis in colorectal cancer (CRC) [147]. USP18 expression poses an increased cellular FAO as a target to fatty acid metabolism in NSCLC [148]. Target hypoxic cancer cells with the combination of β-oxidation inhibitor etomoxir and radiation is proven for anti-lung adenocarcinoma [149]. Long-chain acyl-CoA dehydrogenase (ACADL) as an enzyme that regulates β-oxidation is a promising target for regulating Hippo/YAP pathway to confer anti-tumor imunity [136]. Moreover, interleukin-17 (IL-17A) can stimulate angiogenesis through promoting FAO and thus a potential therapy for angiogenic vascular disorders that lead to tumor progression [150].

### Mevalonate pathway

Mevalonate or HMG-CoA reductase pathway is an essential metabolic pathway in cancers. Ferroptosis, a non-apoptotic regulated cell death (RCD) in cancers, can be regulated through mevalonate pathway. This limits multiple signaling molecules in TME [151]. One of the key enzymes in mevalonate pathways, farnesyl pyrophosphate synthase (FPPS), mediates TGF-β1-induced cell invasion and blocks EMT process. This is mediated via the RhoA/Rock1 pathway [152]. Another rate-limiting enzyme in the mevalonate pathway is hydroxy-3-methylglutaryl coenzyme A (HMG-CoA) reductase (HMGCR). HMGCR as a target for fluvastatin, a statin medicine against cholesterol and cardiovascular diseases, suppressed NSCLC cell growth and induced apoptosis [153]. Moreover, cerivastatin of the mevalonate pathway has anti-cancer activity against ALK tyrosine kinase inhibitors (ALK-TKIs) resistance both in vitro and in vivo. This is evidenced by cytoplasmic retention and inactivation of transcriptional co-regulator in YAP ALK-rearranged lung cancer [154].

A master regulator in mevalonate pathway (MVP), SREBP2 is a novel substrate for USP28, a deubiquitinating enzyme. Silencing of USP28 can limit the expression of MVP enzymes with a lower metabolic flux, and a dual USP28/25 inhibitor reduces viability of LSCC cells [155]. The impairment in mitophagy flux by temozolomide-perillyl alcohol conjugate induces lysosomal dysfunction in NSCLC. This is to some extent depending on downregulation on the small GTPase RAB7A via mevalonate pathway [156]. MiR-122-5p targets p53 thereby obstructing the mevalonate pathway and promote apoptosis in NSCLC [157]. Moreover, HMG-CoA statin/erlotinib co-treatment-mediated cytotoxicity mediates erlotinib resistance in K-ras mutated NSCLC [158].

### Mitochondrial respiration pathway

In tumorigenesis, the mitochondrial bioenergetics, dynamics, and signaling are experimentally evident. Mitochondrial respiration via upregulating OXPHOS fuels tumorigenesis. In NSCLC, increased heme synthesis and uptake generate intense ATP through mitochondrial respiration and thus promote tumorigenic functions. In addition, both mitochondrial fission and fusion play a key role in tumorigenesis, making mitochondria a prospect in energy reprogramming approaches for cancer MR [159]. In NSCLCs, mitochondria-targeted genes include 34 in lung adenocarcinomas (LUAD) and 36 for LUSC [160]. Moreover, mitochondrial protein SMAC/Diablo found in the nucleus is a signature for squamous cell carcinoma (SCC) [161].

Mitochondrial PEP-carboxykinase (PCK2) plays a key role in cancer cell MR via glucose-independent cell growth and metabolic stress resistance in NSCLC [162]. The downstream ERK/P90RSK signaling pathway of TIMM50 (translocase of the inner mitochondrial membrane 50) can enhance the tumor proliferation and invasion of NSCLC via enhancing phosphorylation [163]. With the inhibition of Nrf2 expression and mitochondrial respiratory chain complex in LSCC, (+)-usnic acid can induce ROS-dependent apoptosis and thus a prospective clinical trial for this subtype of NSCLC [164]. The predicted nuclear-mitochondrial cross-talks are associated with the alteration of mitochondrial genes. Among these genes, LC subtype-specific classical molecular signatures is prominent and this potential biomarker can be used in developing therapeutic targets [160]. Through mitochondrial membrane depolarization, the proliferation of NSCLC cells is inhibited by Nisin ZP exposure. While this was observed with increased ROS generation on cell lines, an in vivo follow-up study might lead to therapeutic development for NSCLC [165].

### Arginine pathway

As one of the most versatile amino acids, arginine serves as a precursor to many molecules such as protein [166]. L-Arginine promotes the interaction of T cells with tumor antigens, and L-arginine plays a key role in the survival and progression of arginine auxotrophic tumors [167]. Circulating L-arginine can predict the lifespan of cancer patients undergoing immune checkpoint inhibitor treatment option [168]. The suppression of tumor cell viability by myeloid lineage to deplete arginine by arginase 1 signals the role played by neutrophil lineage cells [169]. Protein arginine methyltransferase 7 (*PRMT7*) overexpression promotes metastasis in NSCLC, and this was predicted to be through the interaction with *HSPA5* and *EEF2* [170]. Through the process of enhancing small cell lung cancer (SCLC) tumor growth, coactivator-associated arginine methyltransferase 1 (CARM1) regulates arginine methylation of Smad7 [171]. Moreover, autophagy inhibitors protect recombinant human arginase (rhArg)-treated NSCLC cells, and thus, rhArg-induced autophagy and apoptosis is anti NSCLC progression [172]. Through influencing arginine synthesis, *Aconiti Radix Cocta* (ARC) is suggested to be an anti-tumor by regulating the energy metabolism that influence arginine synthesis [173].

### Pentose phosphate metabolic pathway

PPP is an essential metabolic pathway that supports the growth and invasion of cancer cells. TP53-induced glycolysis is the main apoptosis regulator (TIGAR) in PPP [174]. MicroRNA (miR)-218 (miR-218) reduced glucose consumption in NSCLC through PPP [175]. PPP-related lncRNAs for NSCLC has an improved detection and treatment based on the different upregulated immune checkpoints in C1 subtype [176]. Moreover, it could identify lncRNA PTTG3P levels associated with cell proliferation NSCLC and thus a new therapeutic and prognostic strategies [177]. PPP-related proteins, NF-E2-related factor 2 (Nrf2) is a prognostic significance and associated with NSCLC histology [178]. The highly oxidative environment of the lung induces controlled stress response pathways. Lung tumors harboring TF nuclear factor erythroid-2-related factor 2 (NFE2L2/NRF2) pathway alterations created questions as to the exploitation of both immune and metabolic features in treating LUSC. It is found that the metabolites identified in the plasma of Keap1^f/f^/Pten^f/f^ tumor mice are associated with reprogramming of the PPP [179].

Through PPP, palbociclib reduces the activity of the limiting enzyme, glucose 6-phosphate dehydrogenase. This may target CDK4/6 inhibition with glutaminase inhibitors for NSCLC patients, especially those with RB-proficient tumors [180]. The functional role and regulatory mechanism of keratin 6A (KRT6A) overexpression can increase PPP flux by upregulating glucose-6-phosphate dehydrogenase (G6PD) levels [181]. Xanthatin can attenuate PPP in chemoresistance to cisplatin (DDP) resistance for lung cancer, and induce increased ROS levels and apoptosis. This mechanism can mitigate the DDP-resistant antioxidative capacity [182]. C-C motif chemokine 18 (CCL18), that is M2-tumor-associated macrophages, regulates post-translational modifications in A549 cells via PPP [183]. Loss of KEAP1, a negative regulator of the antioxidant response transcription factor NFE2L2/NRF2, activates the PPP in KRAS-mutant LUAD cancers [184]. Specific energy reprogramming episodes in lung cancers expression metabolic targeted therapy (Table 1) and the energy reprogramming mechanism are sketch as

**Table 1:** Specific energy reprogramming episodes in lung cancer expression in metabolic targeted therapy

Targeted molecules/genes	Subtypes of lung cancer	Experimental model	Mechanism	Efficiency	Reference
MROS	LCa and SCC	Cell lines	• Increase ○ MMP ○ Intracellular ATP content ○ MROS	• MP may induce radio sensitization	[185]
PFKP	NSCLC	Human tissues	• Decreased ○ Glucose uptake rates ○ Lactate levels ○ ATP concentrations	• PFKP can regulate the level of glycolysis • This is associated with cell proliferation.	[186]
GFPT2	NSCLC	Cell lines	• Upregulated HBP genes, *GFPT2* • Less changes in PPP and TCA cycle	• *GFPT2* as a critical regulator of tumor MR in adenocarcinoma	[187]
mTOR	SCC	Human tissues	• GSK3α/β signaling pathway • Upregulates glutaminolysis	• Broad spectrum of hyper metabolic tumors	[188]
GSTO2	LSCC	Cell lines	• β-Catenin expression • Mitochondrial membrane potential	• The p38/β-catenin signaling pathway.	[189]
GLUT1	SqCC	Cell lines and mice model	• High ^18^F-FDG uptake	• Poor prognostics	[190]
Glycolysis-related gene	LUSC	Human tissues	• 5 glycolysis-related gene	• A novel glycolysis-related gene	[191]
lonidamine	NSCLC	Cell lines	• Varied glycolysis response patterns	• Pathways were not related to histology.	[192]
KDM2B	LUSC	Cell lines, tumor tissues, and mice model	• Reduced ○ Glucose consumption ○ Lactate production ○ ATP level ○ LDHA and GLUT1	• Inactivation of the PI3K/Akt/mTOR	[193]
m^6^A regulator gene	Lung cancers	Human tissues and cell lines	• KIAA1429 • METTL3 • IGF2BP1	• Pathology-specific regulators of m^6^A RNA modification	[194]
*LINE-1-FGGY*	LUSC	Human tissues	• Nevirapine • Efavirenz	• A biomarker and therapeutic target	[65]
Glycolysis-related gene	LUSC	Human tissues	• Glycolysis-related gene signature	• biomarkers for targeted therapy.	[195]
Maackia amurensis	NSCLC	Cell lines	• Intrinsic/mitochondrial pathway	• Adjuvant chemotherapeutic	[196]
	LCa		• Metabolic characteristics and disordered	• Subtyping of lung tumors	[197]
Glutamate	Lung cancer	Clinical trial	• Plasma glutamate • Amino acids • β-Hydroxybutyrate	• Energy-balance-related metabolites	[70]
GLUT1, PCK1 and PCK2	NSCLC	Cell lines and Human tissues	• Hypoxia regulated ○ Glycolysis ○ Gluconeogenesis	• Future therapeutic strategies	[198]
Kinase	NSCLC	Human tissues	• Focal adhesion kinase • C-terminal Src kinase	• Potential molecular biomarkers	[199]
ALDOA	NSCLC	Cell lines	• Reduced ○ Extracellular lactate ○ Nuclear distribution of PKM2 intracellular ATP levels • Elevated extracellular glucose	• EGFR/MAPK pathway is partly modulated	[200]
TMPRSS11B	Human bronchial epithelial cells (HBECs)	Cell lines	• Enhances ○ Lactate export ○ Glycolytic metabolism	• Transformation of immortalized cells	[201]
GLUT1	NSCLC	Retrospective study	• Micropapillary/solid histology lymphovascular invasion • Advanced pTNM stage	• Heterogeneity in patients	[202]
Physcion 8-O-β-glucopyranoside (PG)	Lung cancers	Cell lines and mice model	• Mitochondria-dependent apoptosis ○ miR-21/PTEN/Akt/GSK3β signaling pathway.	• Lung tumor energy utilization	[203]
MTV and TLG	NSCLC	Human cohort study	• High MTV and TLG values as poor prognostic factors	• A heterogeneous disease	[204]
LKB1	NSCLC	Mouse model	• ADC-to-SCC-AST • PPP deregulation and impaired FOA redox imbalance	• Drug resistance	[205]
p53	LSCC	Cell lines	• Inositol 3-phosphate synthase (ISYNA1) ISYNA1 activation • p53 response element in the seventh exon.	• A novel role of p53 in myo-inositol biosynthesis	[206]
ACBP	NSCLC	Cell lines	• Modulating β-oxidation.	• ACBP control lung cancer	[207]
[(η^5^-C_5_Me_4_C_6_H_4_C_6_H_5_)Ir(C^C)Cl]PF_6_ (C1)	NSCLC	Cell lines	• Regulation of lysosomal-mitochondrial dysfunction • Release of cytochrome c	• Caspase-associated apoptosis	[208]
Isoalantolactone	LSC	Cell lines	• Cell cycle arrest at G1 phase • Downregulate Bcl-2 • Upregulate Bax	• Dissipation of MMP and generation of ROS	[209]
GLUTs	LUAD and LUSC	KM plotter database	• Better OS is associated with high expression levels of ○ GLUT10 ○ GLUT12	• Prognostic values of GLUT members	[210]
Honokiol	SCC	In vitro lung model	• Changes in redox status	• Honokiol as a potential chemo preventive agent.	[211]
IFN-γ	NSCLC	Cell lines	• FADD-mediated caspase-8/tBid/mitochondria-dependent pathway	• Antitumor activity of IFN-γ	[212]
REE	NSCLC	Observational study	• REE emerges as an independent prognostic factor	• A prognostic factor in metastatic	[213]
PON1 gene	Lung cancers	Human tissue	• Maintained ATP levels • p53-directed signals. • Targeted glycolysis stimulated • phosphorylation of AMPK-α	• ROS deregulation protecting the mitochondria from dysregulation	[214]

*ADC* adenocarcinoma, *ACBP* acyl-coenzyme A-binding protein, *ALDOA* fructose-bisphosphate aldolase, *AST* transdifferentiation, *FADD FAS-*associated death domain, *FAO* fatty acid β-oxidation, *KM* Kaplan-Meier, *IFN-γ* interferon gamma, *LCa* lung carcinoma, *LSCC* lung small cell carcinoma, *LUSC* lung small carcinoma, *MPP* mitochondrial membrane potential, *MTV* metabolic tumor volume, *MR* metabolic reprogramming, *MROS* mitochondria-derived ROS production, *mTOR* rampamycin, *NSCLC* non-small cell lung carcinoma, *PON1* paraoxonase-1 gene, *PPP* pentose phosphate pathway, *REE* resting energy expenditure, *SqCC* squamous cell carcinoma, *TCA* central carbon metabolism, *PG* physcion 8-O-β-glucopyranoside, *LG* total lesion glycolysis, ^*18*^*F-FDG* fludeoxyglucose F18**,** *[(η*^*5*^*-C*_*5*_*Me*_*4*_*C*_*6*_*H*_*4*_*C*_*6*_*H*_*5*_*)Ir(C^C)Cl]PF*_*6*_ *(C1) N*-heterocyclic carbenes-modified half-sandwich iridium(III) complex (where C^C is a N-heterocyclic carbene ligand)

### Summary of blocking common NSCLC metabolic pathways as anti-NSCLC

**Fig. 2:**
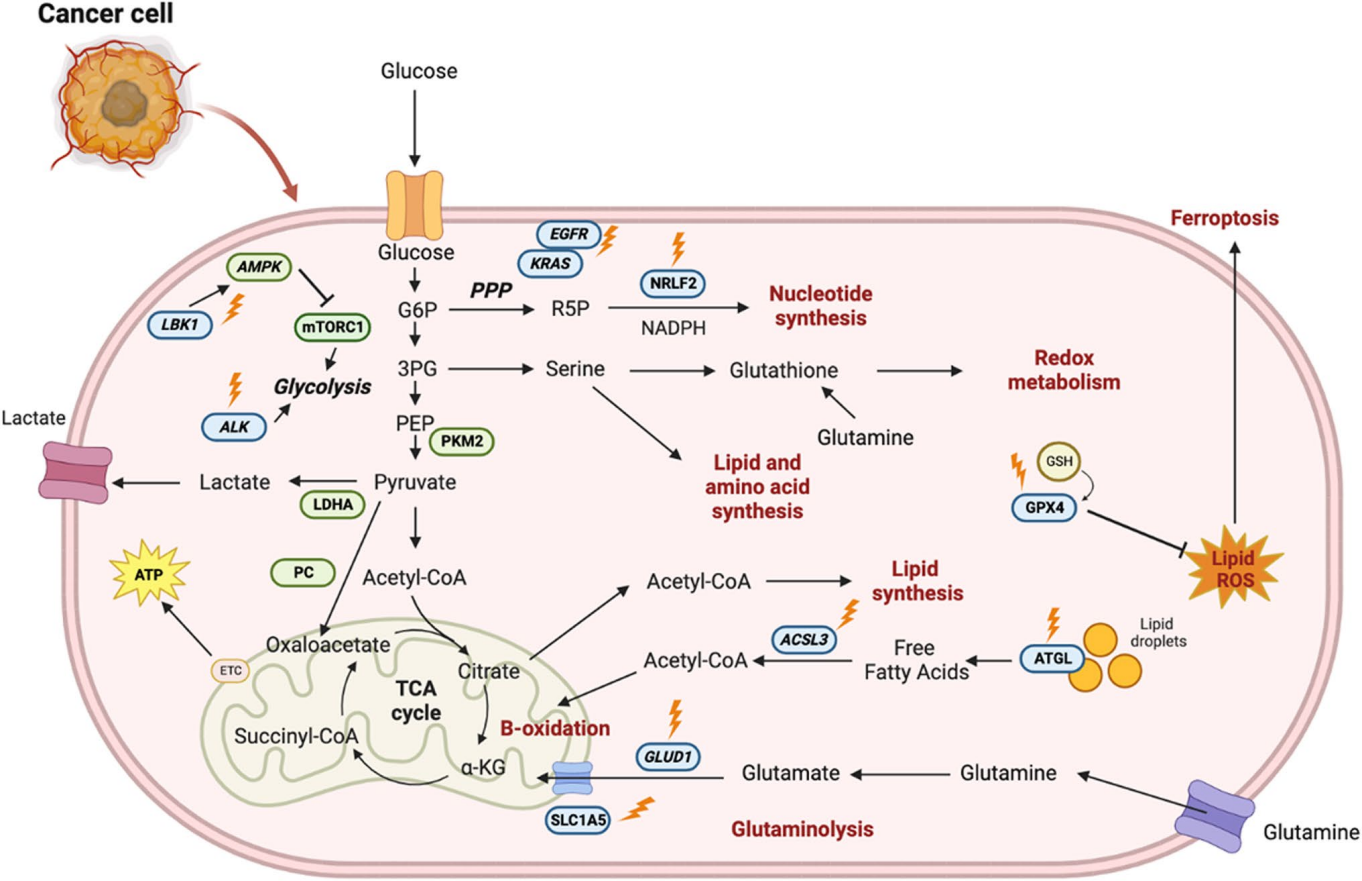
Blocking of common NSCLC metabolic pathways as anti-NSCLC. ACSL3, acyl-coenzyme A (CoA) synthetase long-chain family member 3; AMPK-AMP, protein activated pathway; ATGL, adipose triglyceride lipase; EGFR, estimated glomerular filtration rate; GLUD1, glutamate dehydrogenase 1; FAO, fatty acid oxidation; LDHA, lactate dehydrogenase A; NFE2L2/NRF2, nuclear factor erythroid-2-related factor 2; MGF, phytopharmaceutical mangiferin; PMK2, pyruvate kinase isozymes M2; PPP, pentose phosphate pathway; OSC, osmundacetone

Glutaminolysis is essential for the proliferation of cancer cells, thus inhibiting glutamine transporter SLC1A5 with almonertinib and/or V9302, and downregulating GLUD1 with OSC is a potential therapeutic approach for NSCLC. For lipid biosynthesis, obstructing ATGL activity via prostaglandin E2-independent manners and GPX4 inhibition which can trigger ferroptosis, an iron-dependent form of necrotic cell death marked by oxidative damage to phospholipid are potential energy pathways for NSCLC therapy. In tumor glycolysis pathway, mutant EGFR promotes metabolic reorganization in NSCLC by increasing aerobic glycolysis and PPP, altering pyrimidine biosynthesis, and increasing monounsaturated fatty acid production. When compared to non-malignant cells, KRAS-mutated NSCLC cells produce higher levels of glycolysis enzymes such as PKM2 and LDHA, indicating changes in glucose metabolism and PPP. ALK rearrangements were linked to increased glucose metabolism in highly metastatic adenocarcinoma morphologies. PC, the enzyme responsible for converting pyruvate to oxaloacetate, was shown to be overexpressed and active in NSCLC tumors. Thus, these molecules can be used as therapeutic target for NCSLC. Under metabolic stress conditions, the LKB1-AMPK pathway is activated. The loss of LKB1 expression can alter mitochondrial dysfunction and energy metabolism of the cells, making it an ideal therapeutic target for NSCLC drug designing. For FAO, ACSL3 inhibition with enhanced MGF can inhibit tumor, metastasis, and angiogenesis. PPP-related proteins, Nrf2 is a prognostic significance and associated with NSCLC histology that regulates the cellular defense against toxic and oxidative insults. Its pathway alterations created questions as to the exploitation of both immune and metabolic features in treating LUSC, thus an important target for lung cancer inhibition (Fig. 2).

## Energy metabolism mechanism exerted by cancer drugs used for NSCLC

MA-CLCE downregulates the expression of PI3K/AKT, a survival signaling regulator that modulates Nrf-2 [213]. DSS inhibits phosphorylation of Akt and ERK1/2 and downregulating Nrf2 expression [214]. Another partially by Nrf2 RNAi knockdown was seen with PR-104, a phosphate ester pre-prodrug that regulates the ARE pathway [215]. A1E inhibits the PI3K/Akt and NF-κB survival pathways and induces cytochrome C release and mitochondrial membrane potential collapse [216]. Through the suppression of caveolin-1/AKT/Bad pathway, miR-204 expression sensitizes cisplatin-induced mitochondrial apoptosis [217]. Furthermore, through NF-κB signaling pathways, Euscaphic acid G treatment inhibits IκBα and IKKα/β phosphorylation thus leading to blockage of NF-κB p65 phosphorylation [218]. Bortezomib, a class I histone deacetylase (HDAC) inhibitor prevents the romidepsin-mediated RelA acetylation and NF-κB activation, and this leads to caspase activation [219]. Triptolide involved NF- κB and toll-like receptors and utilizes IL-17 signaling pathway to regulate immune and inflammatory responses thereby promoting apoptosis to inhibit tumor development [220].

Calotropin (M11) pro-apoptotic activity was observed with mitochondrial apoptotic pathway [221]. Similarly, Punica granatum (PLE) as a safe chemotherapeutic agent is also predicted to cause cell cycle arrest via mitochondria-mediated apoptotic pathway [222]. Moreover, through the activation of the intrinsic mitochondrial pathway, CP-1, an extract from the C*oix lachryma-jobi L.* var., can inhibit tumor cell proliferation and induce apoptosis [223]. With mitochondrial signaling pathway, silenced GLIPR1 increases apoptosis [224]. Icariin activates the mitochondrial pathway by inhibiting the activation of the PI3K-Akt pathway-associated kinase, Akt [225]. EELDP triggers apoptosis via the NF-κβ pathway through the increase of the Bax-to-Bcl2 ratio leading to mitochondrial membrane potential fall [226].

Upregulation of ER stress induced unfolded protein response (UPR) pathways with Penfluridol. Moreover, the activation of p38 mitogen-activated protein kinase (MAPK) was a key mechanism for penfluridol-induced autophagosome accumulation [227]. With hematopoiesis (AKT, JAK2, and STAT5), NOV-002 activates c-Jun-NH (2)-kinase, p38, and extracellular signal-regulated kinase [228]. Another Akt/MAPK pathway activation was seen with compound 6q in a ROS-dependent manner to induce apoptosis [229]. Tephrosin can induce cancer cell death via the autophagy pathway [230]. It does this via ROS generation and Hsp90 expression inhibition [231]. Rapamycin and 3-BrPA inhibit mTOR signaling and glycolysis probably due to ATP depletion and reduce expression of GAPDH [232]. Downregulating ALDH3A1 by β-elemene can inhibit glycolysis and enhance OXPHOS, thereby suppressing tumors [233]. Through dose-dependently, Bu-Fei decoction (BFD) can suppress EMT induced by TGF-β1 via attenuating canonical Smad signaling pathway [234]. Downregulating survival with erlotinib can result in reversal of erlotinib resistance in EGFR mutation [235]. Gefitinib and osimertinib effects change in amino acids especially at the tyrosine kinase domain [236]. The energy reprogramming mechanism induced by common anti-cancer drugs for NSCLC is summarized in Table 2.

**Table 2:** The energy reprogramming mechanism induced by common anti-cancer drugs for NSCLC

Anti-cancer drugs	Subtype of NSCLC	Experimental model	Mechanism	Efficiency	Reference
Triptolide	**LUAD**	Human tissue	• NF- κB • Toll-like receptors • IL-17	• Regulation of immune and inflammatory responses and apoptosis	[220]
Penfluridol	**LUAD**	Cell lines and mice model	• Upregulation of ER-UPR • Activation of p38 MARK	• Critical for penfluridol-induced autophagosome accumulation.	[227]
AKR1C3 protein	NSCLC	Patient-derived xenograft models	• Inhibition ○ PI3K/Akt ○ NF-κB • Activated apoptotic intrinsic and extrinsic pathways. • Increased ○ Extrinsic death receptor complex FasL ○ FADD.	• A1E induced MPP collapse and cytochrome C release.	[216]
Hexacyclic triterpene acid	Cisplatin resistant LUAD	Cell lines	• Inducing cell cycle arrest • Apoptosis via NF-κB	• Blockage of NF-κB p65 phosphorylation and nuclear translocation.	[218]
Curry leaves crude extract	NSCLC	Cell lines	• Regulating ○ Different cellular programs ○ Signaling pathways.	• Downregulating PI3K/AKT, which modulates Nrf-2.	[214]
GLIPR1	DDP- LUAD and LCC	Cell lines	• Increase apoptosis ○ Mitochondrial signaling pathway	• Modulation of the response of DDP-resistant	[224]
Caveolin-1	NSCLC	Cell lines	• Downregulation of miR-204 expression • Caveolin-1 overexpression • Suppression of the caveolin-1/AKT/Bad pathway	• Re-sensitize cells to cisplatin-induced mitochondrial apoptosis	[217]
Gefitinib and Osimertinib	NSCLC	Molecular modelling	• Change of amino acids at the tyrosine kinase domain	• Binding energies well correlates with the change in clinical observation.	[236]
Danshensu	LUAD	Cell lines	• Inhibiting ○ Phosphorylation of Akt, ERK1/2 • Downregulating Nrf2 • No effect on HIF-la expression.	• Inhibits tumor cells proliferation in both dose- and time- dependent manner.	[215]
ALDH3A1	LCC and LUAD	Cell lines and mice models	• Enhances glycolysis • Suppresses OXPHOS • Activating the HIF-1α/LDHA	• A new theoretical basis for better clinical applications	[234]
Erlotinib and survivin-shRNA with chloroquine	NSCLC	Cell lines	• Activation of bypass signaling pathway • The changes of TME • Downregulation of survivin	• Reversal of erlotinib resistance in EGFR mutation-positive	[234]
3-BrPA and rapamycin combination	LCC and Lung adenocarcinoma	Cell lines and mice models	• Enhanced antitumor efficacy of 3-BrPA • Inhibition • mTOR • Glycolysis 3-BrPA • ATP depletion • Decreased expression of GAPDH.	• Combinatory preventive effects.	[232]
Icariin activity	LUAD and Lung adenocarcinoma	Cell lines	• Inhibiting of PI3K-Akt pathway-associated kinase, Akt	• Anti-cancer properties without any noticeable toxic effects	[225]
Romidepsin inhibitor	LCC	Cell lines	• NF-κB activation tumor cells. • Prevents • Romidepsin mediated RelA acetylation	• The combined exposure reversed the effects on IκB degradation	[219]
EELDP	LUAD	Cell lines	• EELDP treatment significantly reduced cell migration, wound healing, expression and • Reduced activity ○ MMP-2 ○ MMP-9 ○ NF-κβ	• Apoptosis via the NF-κβ pathway through the increase of the Bax to Bcl_2_ ratio.	[226]
NOV-002	NSCLC	Cell lines	• Increased cell proliferation • Activates ○ p38 ○ c-Jun-NH (2)-kinase ○ Extracellular signal-regulated kinase	• Dose-dependent increase in phosphorylation proteins linked with hematopoiesis (AKT, JAK2, and STAT5)	[228]
Tephrosin	LUAD	Cell lines	• A significant proliferation inhibition in a dose-dependent manner • G (2)/M arrest • ROS generation • Hsp90 expression inhibition.	• Cancer cell death via the autophagy pathway	[231]
PLE	**LUAD**	Cell lines	• PLE is a safe chemotherapeutic agent by • Inhibiting proliferation • Inducing apoptosis • Cell cycle arrest • Impairing cell migration and invasion.	• Apoptosis via MM apoptotic pathway.	[222]
BFD	LUAD	Cell lines and mice models	• Inhibited ○ EMT ○ TGF-β1 ○ Decreasing canonical Smad signaling pathway	• Helps to restrain the malignant phenotype induced.	[233]
Compound 6q	LUAD and LUSC	Cell lines and Mice models	• Induced ROS production in an NQO1 dependent manner • Activated Akt/MAPK pathways in a ROS-dependent fashion	• NQO1-expressing cancer-cell-selective killing property.	[229]
Calotropin	Cisplatin LUAD	Cell lines	• Induced cell cycle arrest at the G2/M phase ○ Downregulating ○ Cyclins ○ CDK1 ○ CDK2 • Upregulating p53 and p21.	• Apoptosis through the MAP.	[221]
CP-1	LUAD	Cell lines	• The activation of the intrinsic mitochondrial pathway	• Anti-tumor effect	[223]
PR-104	LUAD and LCC	Cell lines and mice models	• Partially by Nrf2 RNAi knockdown • Regulation by the ARE pathway.	• Exploitation of tumor hypoxia.	[216]

*ALDH3A1* aldehyde dehydrogenase 3A1, *BFD* Bu-Fei decoction a traditional Chinese herbal formula, *EELDP* ethanolic extract of leaves of *D. pentagyna*, *ER-UPR* endoplasmic reticulum stress-induced unfolded protein response, *EMT* epithelial-mesenchymal transition, *GLIPR1* glioma pathogenesis-related protein 1, *LCC* large cell carcinoma, *MARK* mitogen-activated protein kinase, *MMP* mitochondria membrane potential, *NF-κβ* nuclear factor kappa beta, *NOV-002* a novel glutathione disulfide mimetic, *PGL* Punica granatum (pomegranate, *OXPHOS* oxidative phosphorylation

## Conclusions, expert recommendation, and outlook in the context of 3P medicine

### Phenotyping is crucial for advanced primary and secondary care

In both primary and secondary care, phenotyping is crucial for innovative screening programs, identification of vulnerable subgroups in the population (protection against health-to-desease trasition) and individuals affected by an early stage disease for the targeted energy metabolism reprogramming to protect them against the disease progression. Several clinically relevant phenotypes have been described related to mitochondrial stress and shifted energy metabolism such as the Flammer syndrome phenotype [237] with characteristic symptoms and signs including disturbed microcirculation, psychologic distress, altered sleep patterns, low BMI, low blood pressure, systemic ischemic lesions, low-grade inflammation, shifted metabolic profiles as well as frequently reported increased blood levels of systemic vasoconstrictor endothelin-1 (ET-1), mitochondrial stress, impaired wound healing, pre-metastatic niches, and poor individual outcomes, once FSP carriers are diagnosed with cancers [238]. High ET-1 levels in blood are associated on one hand with the FSP [239] and on the other hand with lung cancer development [20] and poor survival of NSCLC patients [21]. FSP is usually manifested early in life; therefore, there is sufficient room for phenotyping and cost-effective measures to protect FSP carriers against cascading pathologies [238, 240].

Another clinically relevant phenotype is associated with elevated homocysteine (Hcy) levels in blood characterized by either mild or severe hyperhomocysteinemia (HHcy) and compromised mitochondrial health and, synergistically with low folate levels, associated with lung cancer development and progression [24]. Therefore, Hcy metabolism is a promising target for predictive diagnostic and health protective approaches in 3P medicine concepts [241].

Contextually, the quality of mitochondrial health and homeostasis is a reliable target for the predictive approach in overall cancer management

- Beginning with health risk assessment at the stage of reversible damage to the health followed by cost-effective personalized protection against health-to-disease transition (primary care of suboptimal health conditions of individuals predisposed to cancer development)
- Including targeted protection against the disease progression (secondary care of cancer patients against growing primary tumors and metastatic disease) [1, 3].

Health risk assessment utilizing tear fluid analysis as painless and patient-friendly approach for evaluating mitochondria-related biomarkers to predict systemic diseases has been developed and is commercially available [242].

### Breakthroughs on NSCLC energy reprogramming

Inhibiting glutamine transporters, downregulating GLUD1, and knockdown of inhibitors related to glutamine are therpecutive options in energy rewiring treatment options for NSCLC. Obstructing ATGL activity via prostaglandin E2-independent manners, high dose of DEX via M1-like TAMs, and blocking of Nano-DOX-induced PD-L1 via TAM lipid biosynthesis energy reprogramming. PI3K, FGFR1, EGFR, and VEGF/VEGFR signaling and CDK4/6 and KEAP1/NRF2 pathway are key for glycolysis MR in NSCLC. For serine metabolism, LKB1 to LKB1/AMPK signaling and inactivation of *STK11/LKB1* lead anti-tumor efficacy in NSCLC. In FAO, ACSL3 inhibition with enhanced MGF and ACADL regulating Hippo/YAP pathway are anti-tumor immunity strategies. In mevalonate pathway, through the RhoA/Rock1 pathway, FPPS mediates TGF-β1-induced cell invasion and blocks EMT process while inhibiting ERK/P90RSK signaling pathway of TIMM50 and Nrf2 expression induce apoptosis are essential for mitochondrial pathway. While CARM1 regulates arginine methylation of Smad7 in tumor proliferation, rhArg and ARC are essential for MR in the arginine synthesis pathway. PPP-related lncRNAs upregulate immune checkpoints in C1 subtype and identify lncRNA PTTG3P levels in glutaminase inhibitors.

### Limitations

The role of redox-associated genes in the NSCLC pathogenesis and the critical glycolysis-related lncRNAs are not fully explored. Furthermore, tumor DNA methylation data and TCGA-derived miRNA/mRNA sequencing will give a robust energy metabolism for these cancer subtypes. In addition, radiomic features could not identify clinical and core signaling pathways of LUSC, and the EGFR family member of HER3 blocking antibody could not reduce cell and tumor growth. Combination treatments are not explored with regards MR in these tumor subsets. For instance, SLC1A5 inhibition with almonertinib and/or V9302 could be autophagy inhibition-based therapy in NSCLC. Moreover, conventional therapies such as the CPT system are not fully studied. For the resistance phenomenon, metabolic vulnerability of cisplatin-resistant cancers as a target to nucleoside metabolism is not explored at length.

Inhibition of this ATGL activity via high-throughput sequencing the role GPX4 expression to prevent iron-dependent ferroptosis and IL-17A stimulating angiogenesis via promoting FAO angiogenic vascular disorders are new approaches that requires much attention. In addition, NFE2L2/NRF2 pathway alterations on immune and metabolic features in treating LUSC are unclear. Glycolysis flux with low TCA flux and ATP production, ACT therapy, GRGs, and TF regulatory network for NSCLC are not fully studied. In addition, the role of ARC as an anti-tumor by regulating the energy metabolism that influences arginine synthesis is understudied.

### Outlook

In the context of 3PM, MR of NSCLC subtypes has a lot to offer. Although there are some setbacks with regard to establishing biomarkers based on the pathway synthesis, which are highly heterogeneous, there is sufficient room for improvements. For instance, the forms of energy reprogramming studied with various cancers are either monotherapy or combination therapy with limited data output. To this end, the multi-omics approach is expected to provide indication for a robust prediction and targeted treatments. All data must be physiologically evidenced creating reliable patient profiles for treatment algorhithms tailoted to the patient.

(i) Predictive approach

With the MALDI-TOF analysis, the specific proteoforms can predict the patients’ response to ICI therapy for NSCLC based on their intensities of spectral features. In host immunity, proteoform-based diagnostics such as blood-based VeriStrat® proteomic test can accurately predict the response NSCLC patients toward immunotherapy [243]. In complex tumor biology, epithelial cell adhesion molecule (EpCAM) fragment patterns have the potential to reveal cancer-specific changes [244]. The value of validated PEP technology, which is both analytically and robust, will confer efficient diagnosis to NSCLC to explore the source of proteoforms as biomarkers based on its diagnostic potential [245]. Moreover, proteoformic signatures of cancer cellular bioenergetics may serve for prognosis [246].

With proteomic screening, cancer cells switching between energy sources will get stratified between individual subtypes. For instance, the non-glycolysis-related function found a rate-limiting enzyme PFKP as the key regulator in long-chain fatty acid oxidation. This glucose starved-metabolic stress via AMPK pathway will reveal inspirations to other energy sources for tumor growth [247]. The approaches including unsupervised shotgun proteomics with Nanoflow liquid chromatography and high resolution mass spectrometry is capable to identify expressed proteins in relative abundance. This pathway search engine (PSE) may qualify pathways linked to linear energy transfer-induced apoptosis [248] for individualised predictive approach.

(ii) Targeted prevention

The mitochondrial proteomics can reveal invasion abilities in cancers and metastasis, and this has prospects on regulating mitochondrial dynamics [249]. In addition, proteomic analysis is considered a key approach to detect mitochondrial metabolism and energy rewiring thereby preventing the occurrence of metastasis [250]. Based on BMP1 isoforms of NSCLC, the plasma proteoforms revealed distinct differential regulation. Since these isoforms are control-associated, the insights into their mechanism will shed some light on the progress of NSCLC disease progression [251]. The high throughput top-down proteomics (TDP) in an Orbitrap mass spectrometer with its accessible platform will enable proteoforms to be applicable in the preventive medicine [252].

In proteomic analysis, iTRAQ can give isobaric tags for relative, absolute quantitation of mutated genes and TME hypoxia designs for new therapies [253]. When this approach is combined with MALDI-TOF/TOF mass spectrometry analysis and two-dimensional fractionation (OFFGEL/RP nanoLC) could lead not just development of potential treatment options but also biomarker assay for many types of cancers [254]. For instance, with additional data based on proteomics, the study on α-hederin induction of ferroptosis was confirmed to also lead to membrane permeabilization and apoptosis in NSCLC [255]. Protemics has the potential to reveal a number of vulnerable energy stores in biological systems [256]. In addition to dysregulated pathways, proteomic data can reveal cancer associated with adhesion and energy sensing [257].

(iii) Personalized treatments

Protein epitome profiling or epitomics are promising for coprecipitated protein composition and specific posttranslational modification, and while this could classify hypothetical C9 proteoforms in lung cancers, its application is imperative for treatment of NSCLC [258]. The Matrisome DB complete collection data of ECM proteomic will enable the patient to build a comprehensive ECM atlas for targeted therapy [259]. The analysis of proteforms for NSCLC patients after undergoing chemotherapy will reveal plasma protein vitronectin, and this can avert the aftermath consequences [260]. Clinical biobanking and proteoform annotation within chromosome consortia will give an optimal therapeutic strategy for NSCLC [261].

**Fig. 3:**
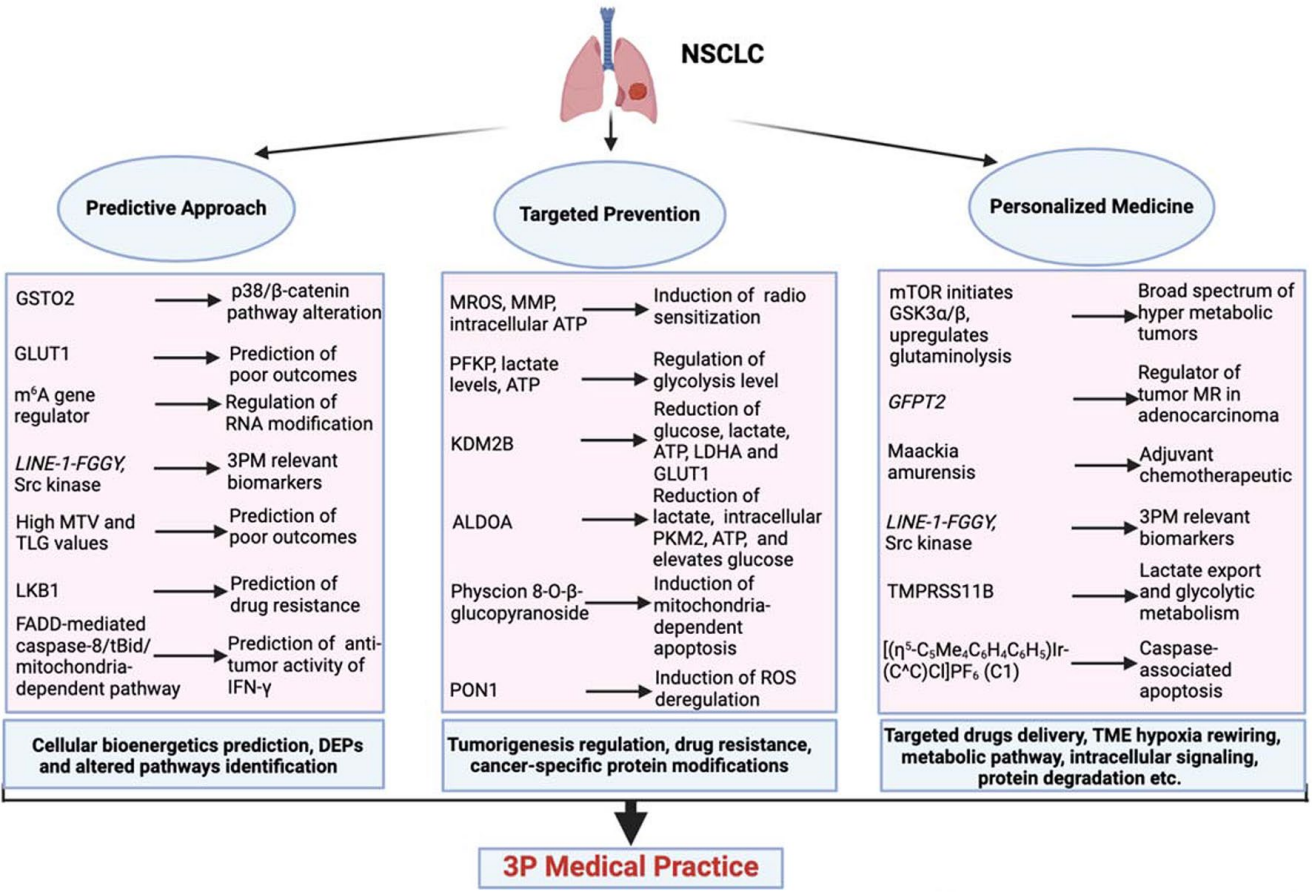
Contributions of energy metabolism pathways to 3PM in NSCLC

In drug delivery, it is imperative for proteomics to adjuvant the metabolic flux analysis. This will give a robust tumor vascular remodeling and initiate blood vessels to deliver the targeted drugs to the needy cells in the system [262]. Proteomic-based screening of resistance biomarker resistance and mechanisms will lead to tailored therapeutic strategies [263], for instance, in identification of exosomes, which are critical for endosomal compartmentalization. A comparative proteomic analysis could give a wholesome of PKM2 especially in cisplatin resistance in NSCLC [264]. The proteostatic regulation and ubiquitination of intramitochondrial proteins have a lot to reveal for drug sensitivity and resistance based on the role of OXPHOS cancers [265]. Two-dimensional electrophoresis (2DE)-based proteoformic approaches reveal metabolic pathway, intracellular signaling cascade, protein degradation, and transcriptional and translational control for cancer progression [266]. Moreover, delta masses at the proteoformic scale identification will decipher the number of glycolytic enzymes and cancer-specific protein modifications for both precision medicine and also for MR therapeutic options [267]. Figure 3 summarizes corresponding innovation and clinical relevance.

### Supplementary information

ESM 1 (DOCX 18 kb)

## Data Availability

No datasets were generated or analysed during the current study.

## Abbreviations

ACADL: Long-chain acyl-CoA dehydrogenase
ACSL3: Acyl-coenzyme A (CoA) synthetase long-chain family member 3
ACT: Adoptive T cell therapy
ALK-TKIs: ALK tyrosine kinase inhibitors (ALK-TKIs)
AML: Acute myeloid leukemia
ANGPTL: Angiopoietin-like protein
ARC: *Aconiti Radix Cocta*
ARE: NRF2-antioxidant response element
ATF3: Activating transcription factor 3
ATGL: Adipose triglyceride lipase
ASPH: Aspartyl β-hydroxylase
BAC: Bronchoalveolar lavage
BFD: Bu-Fei decoction
CAA: Cancer-associated adipocytes
CARM1: Coactivator-associated arginine methyltransferase 1
CCL18: C-C motif chemokine 18
ccRCC: Cell renal cell carcinoma
CD80/86: Cluster of differentiation 80/86
CoA: Acyl-coenzyme A
CPT: Carnitine palmitoyltransferase
CR: Cisplatin resistance
CRC: Colorectal cancer
CTLA-4: Cytotoxic T-lymphocyte antigen-4
DC: Dendritic cells
DCA: Dichloroacetic acid
DEX: High-dose dexamethasone
DNL: De novo lipogenesis
E-BSP: (E)-4-(4-methylbenzyl)-6-styrylpyridazin-3(2H)-one
EMT: Epithelial-mesenchymal transition
ET-1: Endothelin-1
G6PD: Glucose-6-phosphate dehydrogenase
GRG: Explore glycolysis-related genes
FA: Fatty acid
FAO: Fatty acid β-oxidation
FPPS: Farnesyl pyrophosphate synthase
FSCN1: Fascin actin-bundling protein 1
FSP: Flammer syndrome phenotype
FSP1: Ferroptosis suppressor protein 1
GFPT2: Glutamine-fructose-6-phosphate transaminase 2
GPX4: Glutathione peroxidase 4
HBP: Hexosamine biosynthetic pathway
HDAC: Histone deacetylase
Hcy: Homocysteine
HMG-CoA: Hydroxy-3-methylglutaryl coenzyme A
IGFBP-3: Insulin-like growth factor binding protein-3
IGF-I, IGF-II: Insulin-like growth factors I and II
IL-17A: Interleukin-17
KLF2: Kruppel-like factor 2
KLK: KEAP1/NRF2
KRAS: Receptor tyrosine kinases (RTK)-RAS
*LINE-1-FGGY*: Long interspersed element-1
LKBI: Liver kinase B1
KRT6A: Keratin 6A
LCC: Large-cell carcinoma
LMBG: Low-molecular-weight β-glucan
LSCs: Leukemia stem cells
LUAD: Lung adenocarcinoma
LUSC: Lung squamous cell carcinoma
MAPK: Mitogen-activated protein kinase
MDSC: Myeloid-derived suppressor cells
MEMP: Mitochondrial energy metabolic pathway
MGF: Phytopharmaceutical mangiferin
miR-26a: microRNA-26a
MR: Metabolic reprogramming
MTB: Mitochondrial trifunctional protein
MVP: Mevalonate pathway
Nano-DOX: Nanodiamond-doxorubicin conjugates
NFE2L2/NRF2: Nuclear factor erythroid-2-related factor 2
NSCLC: Non-small cell lung cancer
OSC: Osmundacetone
OXPHOS^HI^: Oxidative phosphorylation
PCK2: PEP-carboxykinase
PD-1: Programmed death receptor-1
PD-L1: Programmed death ligand-1
PHGDH: Phosphoglycerate dehydrogenase
PLE: Punica granatum
PPARγ: Peroxisome proliferator-activated receptor gamma
PPP: Pentose phosphate pathway
PRDX: Peroxiredoxin
*PRMT7*: Protein arginine methyltransferase 7
PSE: Pathway search engine
PTHrP: Parathyroid hormone-related protein
PTPRF: Protein tyrosine phosphatase receptor type F
PYGL: Protein glycogen phosphorylase
RCD: Regulated cell death
redox-LPS: Redox-related lncRNA prognostic signature
SCC: Squamous cell carcinoma
SCCA1: Squamous cell carcinoma antigen 1
SCLC: Small-cell lung carcinoma
STK11: Serine/threonine kinase 11
TAMs: Tumor-associated macrophage
TCA: Central carbon metabolism
TCGA: The Cancer Genome Atlas
TF: Transcription factor
TFEB: Transcription factor EB
TIGAR: TP53-induced glycolysis is the main apoptosis regulator
TME: Tumor microenvironment
TRAP1: Tumor necrosis factor receptor-associated protein 1
TTICs: Tumor-infiltrating immune cells
UPR: Unfolded protein response
