# Supplementary material for: Energy metabolism as the hub of advanced non-small cell lung cancer management: a comprehensive view in the framework of predictive, preventive, and personalized medicine
Source: EPMA J. 2024 Apr 8;15(2):289–319. doi: 10.1007/s13167-024-00357-5 (PMC11147999; doi:10.1007/s13167-024-00357-5)
Supplement: Supplementary file 1 — (DOCX 18 kb) [file 13167_2024_357_MOESM1_ESM.docx]

**3PM innovation highlights**

1. **Working hypothesis in the framework of 3P medicine**

With distinct energy programming patterns, 3P medicine has potential therapeutic approaches for NSCLC. Both mitochondrial dysfunction and energy metabolism of the cells will give ideal therapeutic targets for NSCLC drugs designing. The pathway alterations created questions as to the exploration of both immune and metabolic features in treating NSCLC, thus an important target for lung cancer inhibition. For instance, with *autophagy inhibition*, autophagy recycles macromolecules to provide mitochondrial substrates for nucleotide synthesis and energy homeostasis. The 3P medicinal approach has the potential of predicting NSCLC diseases occurrence and can prevent the severe complications. With the predictive response assessment, the early-stage diagnosis will lead to optimal therapy with a robust prognostic assessment.

1. **Innovation towards the predictive approach, targeted prevention and personalisation of medical services**

Predictive approach: The cellular bioenergetics can be predicted by proteomics signatures of cancer. iTRAQ, MALDI-TOF/TOF and OFFGEL/RP nanoLC could lend biomarker assay. Unsupervised shotgun proteomics with nanoflow liquid chromatography and high-resolution mass spectrometry can identify expressed proteins in relative abundance. This pathway search engine (PSE) will decipher the number of pathway synthesis that are yet to be biologically connected through linear energy transfer-induced apoptosis. Proteoforms has the potential to reveal a number of vulnerable energy stores in biological systems.

Targeted Prevention: Mitochondrial proteomics dynamics can regulate tumorigenesis and metastatic. Proteomics-based screening lead to identification of resistance biomarkers and their mechanisms. The proteostatic regulation and ubiquitination of intramitochondrial proteins have a lot to reveal for drug sensitivity and resistance based on the role of OXPHOS cancers. Delta masses at the proteoformic scale identification will decipher the number of glycolytic enzymes and cancer-specific protein modifications for both precision medicine and also for MR therapeutic options.

Personalised Medicine: In drug delivery the metabolic flux analysis will give a robust tumor vascular remodeling and initiate blood vessels to deliver the targeted drugs. iTRAQ can reveals mutated genes and help in rewiring TME hypoxia. Two-dimensional electrophoresis (2DE)-based proteomic approaches reveal metabolic pathway, intracellular signaling cascade, protein degradation, transcriptional and translational control for cancer progression. In dysregulated pathways, proteomics data can reveal cancer associated with adhesion and energy sensing thus, prevent the tumor proliferation.

1. **How does the presented innovation go beyond the state of the art contributing to the paradigm shift from reactive medicine to PPPM?**

Presented metabolic phenotyping is instrumental for innovative population screening, health risk assessment, predictive multi-level diagnostics, targeted prevention and treatment algorithms tailored to personalized patient profiles – all are essential pillars in the paradigm change from reactive medical services to 3PM approach in overall management of lung cancers. This article highlights 3PM relevant innovation focused on the energy metabolism as the hub to advance NSCLC management benefiting vulnerable subpopulations, affected patients and healthcare at large.
